# The Middlesex Hospital

**Published:** 1894-12-08

**Authors:** 


### THE MIDDLESEX HOSPITAL.

Public interest in our hospitals has never been greater than at present. Visitors to a hospital are, however, chiefly concerned with the wards and their inmates, and practical arrangements of the interior. Of the important organisation which administers the whole of one of our large metropolitan hospitals the community at large are entirely ignorant. There is a shadowy knowledge of the office of chairman at a hospital, but what his duties are, or how he comes in contact with the practical management of the institution, very few even pretend to know. Thus few, and more's the pity, who give to our medical charities are able to discriminate between a well and an indifferently organised administration. Practically, the difference is of great importance, as the comfort and health of the patients and the staff, the economical use of funds, and the efficiency of the whole work are affected; and were the governors and other donors to hospitals in a position of knowledge, they would insist upon a really good scheme of management. The voluntary system admits of a standard of efficiency being attained. It is only a little more education of a special kind which is required. Nothing is more useful than a good example, and it is a privilege of which we delight to avail ourselves to bring such into notice, as the quarterly report of the Middlesex Hospital, recently presented, now gives us an opportunity of doing. In a well-administered hospital, be it understood, a board sits weekly, to which the practical points of management are referred, and which conducts the business generally of the institution. This board reports quarterly to a meeting of the governors. One of these quarterly meetings has just taken place at the Middlesex Hospital, and it is the general excellence of administration which is revealed by the report presented on that occasion, and to the able manner in which it is set forth, that we would especially draw our readers' attention. The report is a printed one; thus it can be extensively circulated amongst those who are already helpers of the institution, and be the means of gaining new interest and new friends.

A list of the names of those present together with a short summary of proceedings at the meeting is frequently all that can be gleaned from the reports that reach the public. This does not help those who are interested in the progress and work of the hospital. Mr. Melhardo has given us a better opportunity. By studying his report we see with what care, ability, and interest the weekly board have discharged their duties; duties so varied and numerous as to prove that only those whose heart is in the work would undertake an office which demanded such a call upon time and energy. We are told that a new operating theatre is advancing towards completion; so is a convalescent home at Clacton-on-Sea. The appeal for funds to erect a new wing for female cancer patients is meeting with good response. Another paragraph shows the wisdom of the board in recognising some undesirable points in the arrangements for patients whereby waste was incurred, and promptly remedying the defect. That the needs of the nursing staff are not overlooked is apparent also, as a nurse who has done good service for the hospital has received a substantial recognition of her services on retiring through ill-health. We are glad to learn that friends have not forgotten the Middlesex in the matter of donations and legacies. The largest sum received through the latter source was £5,000, from a late governor of the hospital, Mrs. Matilda Pilcher. Welcome and important as legacies undoubtedly are, they are frequently sources of great difficulty to the management before they can be secured, and the report before us shows a strong instance of this. At the meeting to whom the interesting report was read, some points of which we have summarised, Mr. H. Custance, secretary of the Metropolitan Hospital Sunday Fund, who was formerly the secretary-superintendent, and is now a governor of the hospital, was unanimously voted to the chair. Those present included Lord Sandhurst, Chairman of the weekly board, Sir Lionel Pilkington, Bart., vice-president, and the President of the Royal College of Surgeons.

